# The Heart Failure Epidemic

**DOI:** 10.3390/ijerph7041807

**Published:** 2010-04-19

**Authors:** Véronique L. Roger

**Affiliations:** Division of Cardiovascular Diseases, Department of Internal Medicine and the Department of Health Sciences Research, Mayo Clinic, Rochester, MN 55905, USA; E-Mail: roger.veronique@mayo.edu; Tel.: +1-507-538-6916; Fax: +1-507-284-1516

**Keywords:** epidemiology, heart failure, population studies

## Abstract

Heart failure has been singled out as an emerging epidemic, which could be the result of increased incidence and/or increased survival leading to increased prevalence. Knowledge of the responsibility of each factor in the genesis of the epidemic is crucial for prevention. Population-based studies have shown that, over time, the incidence of heart failure remained overall stable, while survival improved. Therefore, the heart failure epidemic is chiefly one of hospitalizations. Data on temporal trends in the incidence and prevalence of heart failure according to ejection fraction and how it may have changed over time are needed while interventions should focus on reducing the burden of hospitalizations in hear failure.

## 1. Heart Failure: An Epidemic in Need of Investigation

Heart failure has been singled out as an emerging epidemic [1]. The Merriam-Webster dictionary defines epidemic as “an outbreak or product of sudden rapid spread, growth, or development; *specifically*: a natural population suddenly and greatly enlarged.” This may be occurring as a result of increased incidence, increased survival leading to increased prevalence or both factors combined. Knowledge of the respective responsibility of each of these factors in the genesis of the heart failure epidemic is crucial for prevention strategies. To identify studies for this review, we searched the Medline database for literature with the subject headings “heart failure, epidemiology prevalence, incidence, trends” between 2005 and present. We identified 612 articles from which we selected the ones that form the basis of this article after reviewing the abstracts for relevance to the topic.

## 2. Classification and Definitions

The 2005 American Heart Association/American College of Cardiology guidelines [2] define heart failure as “a complex clinical syndrome that can result from any structural or functional cardiac disorder that impairs the ability of the ventricle to fill or eject blood”. The guidelines underscore that “it is largely a clinical diagnosis that is based on a careful history and physical examination”. These statements emphasize that heart failure is a syndrome and not a disease that that its diagnosis, which relies on a clinical evaluation, is quite challenging. To investigate the heart failure epidemic, standardized criteria that can be used on a large scale for ascertainment through medical records are needed.

### 2.1. Presence of Heart Failure

Several criteria have been proposed, including the Framingham criteria [3], the Boston criteria [4], the Gothenburg criteria [5] and the European Society of Cardiology criteria [6]. As shown in Table 1, all rely on similar indicators of symptoms and elevated filling pressures and combine data from the medical history, physical examination and chest X-ray.

It is important to note that the European Society of Cardiology criteria [6] require objective evidence of cardiac dysfunction. This is feasible in population sciences solely when uniformly used, which is still infrequently the case even in contemporary practice [7–10]. When the Boston and Framingham criteria were compared against the masked assessment of a cardiologist [11], their sensitivity was excellent at 100%. The specificity of the Framingham score and its positive predictive value were lower than those of the Boston score for definite heart failure, but it provided greater sensitivity to diagnose possible heart failure. Altogether, five of the six scores studied by Mosterd *et al.* [11] were broadly similar for the detection of heart failure. It should be noted however that the sample size was small thereby limiting the ability to detect differences across scores. Some authors recommended the use of the Boston criteria over other criteria for the diagnosis of HF in older adults due to their construct validity and improved prediction of adverse outcomes [12]. The Cardiovascular Health Study criteria rely on a panel of physicians that assign a diagnosis of heart failure by reviewing data on history, physical examination, chest radiogram report and medication use [13]. The comparison of the Framingham criteria to the Cardiovascular Health Study criteria yielded similar results [13]. Altogether, the Framingham criteria offer good performance. As they are unaffected by time and use of diagnostic tests, they are well suited for studies of secular trends. Clinical cases of heart failure not meeting validation criteria are also important to capture in studies as they contribute to the epidemic and to use of health care resources.

### 2.2. Systolic and Diastolic Heart Failure

Once the diagnosis of heart failure is established, further classification requires the understanding of the parameters of left ventricular function to understand the physiology of the heart failure syndrome in each patient.

The first step is the assessment of left ventricular ejection fraction to classify heart failure into heart failure with preserved or reduced ejection fraction.

While a reduced ejection fraction identifies systolic heart failure, different thresholds to define preserved ejection fraction have been recommended by different groups as shown in Table 2. In the Framingham Heart Study [14] and Olmsted County Study [15–19], the cut-off of 50% is used as recommended by the American Heart Association and American College of Cardiology guidelines [20]. While 55% was recommended in the recent American Society of Echocardiography guidelines [21], 50% remains the most commonly used cut-off and is recommended for use until there are more data to support another limit [22,23]. It is important to be mindful in this context of the arbitrary nature of such a threshold and of the variability of imaging studies that enable to determine this cut-point [24].

Conversely, heart failure with an ejection fraction of 50% or greater in the absence of major valve disease defines heart failure with preserved systolic function [14]. Using this threshold, ejection fraction is preserved in more than half of heart failure cases in the community [25,26]. To further classify subjects with heart failure and preserved ejection fraction, several criteria have been proposed [14,22,27], and the need to assess diastolic function with catheterization *versus* echocardiography-Doppler to define diastolic heart failure remains controversial [14,22,23,28–30].

Invasive measurements with conductance catheters have historically been considered the gold standard to measure filling pressures [31]. However, as with all invasive approaches, it carries intrinsic risks, is seldom used in practice [10] and is not feasible for population studies. While magnetic resonance imaging (MRI) is an excellent tool to assess cardiac volumes and mass [32], its use to evaluate diastolic function is presently not established [33,34]. Thus, echocardiography-Doppler is currently the most feasible approach to assess diastolic function. Its results will likely have the most relevance to contemporary practice, as echocardiography-Doppler examination is a Class I indication (“conditions for which there is evidence and/or general agreement that a given procedure or treatment is beneficial…”) in the heart failure guidelines [2] and left ventricular function assessment is a core performance measure for heart failure under the Joint Commission on Accreditation of Heath Care Organizations (JCAHO) [35]. In the past, Doppler indices for diastolic function have been criticized for their complexity, dependency on loading conditions and limited reproducibility [23,36,37]. The development and validation of Tissue Doppler Imaging (TDI) has been an important advance which, combined with mitral inflow measurements, provides a feasible and reproducible approach to assess filling pressures [23,38–45]. These techniques enable classifying diastolic function into mutually exclusive categories, indicative of progressive elevation of filling pressures [26]. Several algorithms have been proposed including recently by the American Society of Echocardiography [46]. The distinction between the existing sets of criteria should not obscure the fact that the basic measurements are similar such that it is important for the user to select the algorithm that he/she is most comfortable with based on the performance of the laboratory where the measurements are to be performed.

Regardless of the measurements consideration, the mechanistic link between the elevation of filling pressures and the disease process is also controversial. Indeed, the causal role of intrinsic diastolic dysfunction (impaired relaxation and increased diastolic stiffness) [28] was challenged against that of altered ventricular-vascular coupling [47–49]. As underscored [48,50], the altered ventricular-vascular coupling hypothesis needs to be considered cautiously as heart failure with normal ejection fraction is likely a heterogeneous entity within the heart failure syndrome. Further, evaluating the putative role of other mechanisms requires complex measures that cannot be easily implemented in large scale epidemiology studies. One additional issue that must be considered is pulmonary hypertension. Pulmonary hypertension is frequent and often severe in heart failure with preserved ejection fraction. Although pulmonary venous hypertension contributes to pulmonary arterial hypertension, it does not always fully account for its severity, suggesting that a component of pulmonary arterial hypertension also plays a role [51]. Importantly, these mechanisms are not exclusive of one another and measuring diastolic function as can be done by echocardiography- Doppler is an important step towards improving our understanding of the heart failure syndrome [52].

## 3. Incidence and Prevalence of Heart Failure

As measured by Vital Statistics, the burden of heart failure and its societal cost are enormous, thereby epitomizing a public health problem as underscored in guidelines from the American Heart Association and American College of Cardiology [2]. Indeed, heart failure is the single most frequent cause of hospitalization in persons 65 years of age or older, and approximately 4.9 million Americans carry this diagnosis [53]. National Hospital Discharge Survey data from 1979 to 2004 indicate that the number of hospitalizations with any mention of heart failure tripled from 1,274,000 in 1979 to 3,860,000 in 2004 [54]. As hospitalization statistics are event-based, not person-based, this allows multiple hospitalizations for the same individual to be counted without distinguishing between first and subsequent admission such that incidence cannot be derived from such data. Data on the prevalence and particularly the incidence of heart failure are relatively sparse and lack consistency as shown in Table 3. Several observations can be made from the review of this table. Firstly, several estimates are derived from hospital discharges. Further, the diagnoses are not always validated using standardized criteria, and shifts in hospital discharge diagnoses preferences after the introduction of the Diagnosis-Related Groups payment systems have been documented [55,56]. For heart failure in particular, the potential for “upcoding” of discharge diagnoses due to reimbursement incentives is well documented [57]. Thus, national statistics and claims data are inadequate to assess the burden of heart failure. Secondly, inpatient data may not capture all cases of heart failure as care is increasingly delivered in the outpatient setting [58]. Studies using surveys of physicians or self-report are by design more inclusive in their ascertainment. They reported relatively broad ranges of prevalence without validation. When validation was carried out, approaches have ranged from medical record review and adjudication as in the Cardiovascular Health Study [59] to the use of criteria such as the Framingham, Boston, or European Society of Cardiology criteria [3,4,60]. Using standardized criteria, the incidence of heart failure in an earlier study from Framingham was between 1.4 and 2.3 per 1000/year among persons 29 to 79 years old [3]. However, the size of the cohort inherently limits power to analyze secular trends in this report. Among the studies of secular trends [61–66], few [61,65,67,68] included outpatient data. Others used hospitalized cases without validation, and are thus subject to secular changes in hospitalization practices and coding patterns, which likely confound time trends in incidence. It should not be surprising therefore that their results differ. Croft [62], comparing the rates of initial hospitalization for heart failure using Medicare hospital claims in 1986 and 1993, reported an increase in the initial hospitalization for heart failure, while acknowledging limitations related to the lack of validation and possible incomplete ascertainment of incidence. Conversely, Stewart [63,64] suggested that the heart failure epidemic, as measured by trends in hospitalization in Scotland in the 1990s, had “leveled off”. These data suffer from similar limitations, *i.e*., lack of validation and sole use of inpatient data. They, however, prompt the question of whether the stabilization of the heart failure hospitalization rates could be offset by increasing out patient care practice. Data from the Henry Ford Health system, a managed care organization [65], indicated that the prevalence of heart failure was increasing over time but did not detect any secular change in the incidence or mortality of heart failure. Reports from the Framingham Heart Study [68] and the Olmsted County Study [67] indicate that when outpatient heart failure is included, as is the case in these population-based studies, over time the incidence of heart failure remained stable [67] or even declined in women [68]. It should be noted that, while the interpretation and informal comparison of trends across studies is appropriate, as adjustment approaches differ, the absolute numbers cannot be compared. Importantly, the trends noted among the elderly are different underscoring the importance of careful consideration of age in the evaluation of the burden of heart failure. Indeed, data from the Kaiser Permanente system comparing the incidence of heart failure in 1970 to 1974 and 1990 to 1994 among persons 65 years old or greater indicated that the age-adjusted incidence increased by 14% over time and was greater for older persons and for men [69]. The Framingham and Olmsted County studies have shown trends toward increasing heart failure incidence among older persons contrasting with trends among younger persons. This pattern of increasing trends among older persons in three carefully conducted population studies is important to note given the aging of the population. While these reports provided needed insights into the heart failure epidemic, most studies pertain to white subjects, and data on the burden of heart failure in diverse populations are urgently needed. This underscores the imperative for continued community surveillance of heart failure in diverse populations [57,70]. Data on the incidence and prevalence of heart failure according to ejection fraction and how it may have changed over time are very limited. The available evidence suggests that the prevalence of heart failure with preserved ejection fraction increased over time [15].

## 4. Mortality of Heart Failure

The prognosis of heart failure is poor with reported survival estimates of 50% and 10% at 5 and 10 years, respectively [71–73], and left ventricular dysfunction is associated with an increase in the risk of sudden death [74]. Few population-based data are available on secular trends in the prognosis of heart failure. In Framingham and Olmsted County, earlier studies reported no improvement in the survival of heart failure validated using Framingham criteria [61,75]. More recently, improvement in the survival of hospitalized heart failure among the Scottish population was reported [71] with notable age and sex differences in the magnitude of the secular trends. Several explanations can be offered for these inconsistencies. Firstly, the data reported by McIntyre pertain to more recent years and may, as suggested by the authors, reflect in part the effectiveness of angiotensin converting enzyme inhibitors. However, the median survival improved relatively modestly from 1.2 to 1.6 years such that, while the large sample size (66,547 patients) results in high statistical significance, the clinical significance of this improvement in survival is more modest. Further, as acknowledged by the authors, the analyses relied solely on hospitalized cases, not validated, such that the improvement in outcome may be confounded by trends in coding practice and shifting of hospitalization thresholds.

Regardless, these data resonate with clinical trial data that indicated that angiotensin converting enzyme inhibitors, while associated with large reductions in the relative risk of mortality, resulted in more modest (5.7%) absolute event-rate difference. [76]

Conversely, the administrative data from the Henry Ford Health system, which include outpatient encounters, reported a median survival of 4.2 years without any discernible improvement over time [65]. These large discrepancies in survival estimates underscore the challenges in investigating the heart failure epidemic and help delineate key requirements for such evaluation. This investigation should include all cases of heart failure in a geographically defined population and use standardized validation criteria in order to generate valid longitudinal trends. These analyses should examine trends in hospital admission as an additional outcome as high hospital admission rates after diagnosis provide insights into the outcome of heart failure, independently of disease severity [77–81], and are an important component of its public health burden. Despite notable improvements in survival over time, data from Framingham [68] and Olmsted County [67] underscored the persistently high mortality of heart failure even in more contemporary times: indeed, after age adjustment, estimated 5-year mortality rates were 59% in men and 45% in women during the time period 1990–1999 in Framingham and 50% in men and 46% in women during the time period 1996–2000 in Olmsted County. Improvements in survival were also noted more specifically within an elderly population as shown by data from the Kaiser Permanente system. Indeed, over the two decades between the mid 1970’s and mid 1990’s after adjustment for age and comorbidities, survival after the diagnosis of heart failure improved by 33% in men and 24% in women [69]. Importantly, in the Kaiser Permanente study, improvement in survival was primarily associated with beta blocker treatment. Altogether, these data on mortality coincide temporally with major changes in the treatment of heart failure and thus reflect two key points; heart failure treatment is effective in the community but much progress remains to be accomplished.

As discussed above, the data available suggest that the proportion of heart failure with preserved ejection fraction increased over time. As the survival of heart failure with preserved ejection fraction remained unchanged [16], its prevalence can be assumed to be increasing thereby underscoring the growing importance of heart failure with preserved ejection fraction as a public health problem and the urgent need to define specific treatment for this entity [15].

### 4.1. Cause of Death in HF

While the causes of death in heart failure can be challenging to ascertain, there is evidence that, in the community and within the context of stable overall survival, cardiovascular deaths were less frequent among subjects with preserved ejection fraction. Indeed, in Olmsted County, Minnesota, among 1063 persons with heart failure, the leading cause of death in subjects with preserved ejection fraction was non-cardiovascular (49%) *versus* coronary disease (43%) for subjects with reduced ejection fraction. The proportion of cardiovascular deaths decreased from 69% in 1979–1984 to 40% in 1997–2002 (p = 0.007) among subjects with preserved ejection fraction contrasting with a modest change among those with reduced ejection fraction (77% to 64%, p = 0.08) [77]. The shift in the distribution of the causes of death towards less cardiovascular causes has important implications for the understanding of secular trends in heart failure, and for therapeutic trials for this condition.

## 5. Hospital Admissions in Heart Failure

As reviewed above, there is evidence that the incidence of heart failure has remained stable over the past 2 decades while survival has improved [65,67,68]. These findings indicate that the heart failure can be conceptualized as a large chronic disease epidemic with an increase in prevalence related to the aging of the population and the improved survival of patients with heart failure [65]. Both factors increase the number of candidates for hospital admissions. Given these epidemiological trends, it should not come as a surprise that, heart failure is the single most frequent cause of hospitalization in persons 65 years of age or older, and that hospital discharges for heart failure increased 157 percent between 1979 and 2002 [82]. These staggering numbers underscore the public health burden of heart failure as highlighted in the guidelines from the American Heart Association and American College of Cardiology [2]. As hospital admissions are the major driver of health care costs in heart failure [83], understanding the epidemiology of hospital admissions in heart failure, its determinants and significance for the outcome of the disease as assessed by the proportion specifically related to heart failure exacerbation is a necessity.

As hospital admissions are event-based, this allows multiple hospitalizations for the same individual to be counted. Thus, this information, while crucial to assess the health care implications of heart failure, does not measure hospitalizations experienced by individual patients. Further, in administrative data, the diagnoses are not validated using standardized criteria, and shifts in hospital discharge diagnoses preferences after the introduction of the Diagnosis-Related Groups payment systems have been demonstrated [55,56]. As mentioned above, for heart failure in particular, “upcoding” of discharge diagnoses related to reimbursement issues is well documented and quite large [57]. Thus, national statistics and claims data do not provide insight into the number of hospitalizations experienced by individual patients living with heart failure and how it may have changed over time. This is important as intense treatment efforts (medications, device and disease management-based) are directed at reducing hospitalizations in heart failure, yet their effectiveness in the population remains to be documented by demonstrating a reduction in admissions over time. Moreover, heart failure is a chronic disease characterized by bouts of exacerbation leading to recurrent hospitalizations. Thus, measures of the frequency of heart failure-specific hospitalizations are essential to gain insight into the effectiveness of its treatment. Indeed, medications for heart failure cannot be expected to appreciably reduce all hospitalizations among persons with heart failure, given the high prevalence of comorbidity in these patients. Despite the importance of these issues, data on the frequency of hospital admissions among subjects with heart failure are relatively sparse and often incomplete as summarized in Table 4. Several observations stem from the review of this table. Firstly, there is little data, contrasting with the perceived magnitude of the problem. Studies are heterogeneous in many ways including setting, population studied, and criteria used to diagnose the index heart failure, and heart failure-related hospitalizations, which seldom included validation. Thus, not unexpectedly, their results lack consistency.

All studies pertain to hospitalized cases and measure re-admissions. Yet, a large proportion of incident heart failure cases are diagnosed in outpatient settings such that the numbers reported do not pertain to the entire spectrum of patients with heart failure [67]. Further, as these reports did not ascertain the incident status of heart failure, their results are affected by incidence prevalence bias, which limits their validity. Indeed, information from an incidence cohort is essential as the outcome of a disease cannot be interpreted if subjects at various stages in their evolution are combined. Despite the intuitive aspect of this point, this is not adequately addressed in previous publications. Additionally, few studies assessed secular trends, which is important given therapeutic efforts to improve survival and reduce hospitalizations in heart failure [84]. Those that did reported conflicting data. Moreover, the impact of death was not taken into account in these studies, which further hinders the validity of their results. Indeed, not accounting for the impact of death will lead to biased results by overestimating the incidence of non-fatal events in a population for which the death rate is higher than the general population as is the case for heart failure [85,86]. Importantly, data on heart failure-specific hospitalizations as opposed to all-cause hospitalizations are even sparser but suggest that heart failure-specific hospitalizations may be noticeably less frequent. To this end, National Hospital Discharge Survey data from 1979 to 2004 indicate that whereas heart failure was the first-listed diagnosis for 30% to 35% of these hospitalizations, the proportion with respiratory diseases and noncardiovascular, nonrespiratory diseases as the first-listed diagnoses increased [54]. In the community of Olmsted County, among a random sample of all incident HF cases in Olmsted County, Minnesota, from 1987 to 2006, hospitalizations were common after HF diagnosis, with 83% of the patients hospitalized at least once. The reason for hospitalization was HF in 17% of hospitalizations and other cardiovascular in 22%, whereas 62% were non-cardiovascular [87]. It is important to characterize recurrent outcomes, like hospitalizations, in chronic diseases like heart failure, the outcome of which is characterized by recurrence/exacerbation. While the analysis of multiple events presents methodological challenges related in part to the correlation of these events, statistical techniques have been developed to address them [88–91]. These have the potential of providing new insight on the outcome of heart failure by characterizing patterns of hospitalizations and identifying subjects at risk for recurrent hospitalizations that should be offered aggressive preventive strategies.

## 6. Etiology of Heart Failure—An Ongoing Controversy

The guidelines underscore the challenge of assigning a cause to heart failure [2]. The etiology of heart failure is a complex issue and it should be approached while focusing on clinically ascertained risk factors while acknowledging that putative causes of heart failure often co-exist and interact. From a public health and prevention perspective, the determination of the prevalence of each respective cause as ascertained clinically is important because of the resulting clinical implications. For example, demonstrating an increase in the attributable risk of diabetes mellitus independently of clinical coronary disease would then prompt further investigations about the mechanisms whereby diabetes leads to heart failure in the absence of clinical coronary disease. Such mechanisms may include occult coronary disease, but within the appropriate analytical framework, would be distinct from clinically established coronary disease.

The etiology of heart failure and how it may have changed over time is not defined. It is conceivable that the increasing burden of heart failure as measured by hospital admissions relates in part to changing etiology, the analyses of which should thus be part of the investigation of the heart failure epidemic and integrated with the analysis of coronary disease and hypertension trends.

While research has focused on coronary disease and hypertension as the etiology of heart failure [1], the obvious importance of defining the respective contribution of these two entities contrasts with the lack of knowledge in this regard. Moreover, the reported data are conflicting and secular trends have infrequently been examined. Yet, the population burden of putative risk factors for heart failure is changing in the population, such that the attributable risk of these risk factors for heart failure may change over time. Understanding the attributable risk of risk factors for heart failure and how it changes over time is crucial for prevention.

Estimates of the prevalence of coronary disease in studies of heart failure vary considerably. Fox [92], using angiography, concluded that coronary disease was causal in 52% of new heart failure cases under age 75 in a geographically defined population and that clinical assessment without angiography under-estimates the contribution of coronary disease to heart failure. However, few patients were over 75 years of age and only 73% underwent angiography. Thus, the inference from these data is limited by selection bias. Reviewing randomized trial data, Gheorgiade concluded the prevalence of coronary disease in heart failure was 68% [93]. However, important methodological considerations limit the inference that can be drawn form these data. Indeed, the limitations in external validity inherent to clinical trials may be even more apparent in heart failure trials, which typically include younger patients and more men than the general population of heart failure [94,95]. Furthermore, entry criteria in heart failure trials are heterogeneous and seldom validated [96]. Finally, heart failure trials often require systolic left ventricular dysfunction [94], thereby excluding a substantial proportion of heart failure cases [16,97]. Reviewing observational reports of patients with heart failure, Teerlink concluded that the prevalence of coronary disease in heart failure was 50% [98], and in yet another study, which was population-based in England and relied on a panel of physicians, Cowie reported that coronary disease was the etiology of heart failure in 36% of the cases [99]. These large discrepancies may reflect, in part, differences in populations and design. More importantly, they underscore our limited knowledge with regards to the etiology of heart failure, which hinders prevention.

Data from the first National Health and Nutrition Examination Survey (NHANES I) indicate that coronary disease had the largest population attributable risk for heart failure at 62% compared to the other risk factors analyzed (hypertension, obesity, diabetes and smoking) [100]. The attributable risk of hypertension was 10% and that of diabetes was 3% due to its low prevalence. This likely underestimates, as acknowledged by the authors, the role of diabetes which was ascertained by self-report among patients enrolled more than 20 years ago with the incidence of diabetes mellitus increasing over time. In the Cardiovascular Health Study, the attributable risk of coronary disease and hypertension for heart failure were similar, between 12 and 13%, with a notable attributable risk of 8% for diabetes [101]. The Framingham Heart Study traditionally underscored a large contribution of hypertension to heart failure [102–105]. More recently, however, it suggested a 41% increase in the prevalence of coronary disease and a 10% decrease in that of hypertension in heart failure [106]. Whether the results of Framingham are generalizable to larger populations remains to be addressed, particularly in light of hypertension trends in the US and in Olmsted County discussed below. Therefore, whether the etiology of heart failure shifted from hypertension to coronary disease remains to be determined. To this end, when the contribution of coronary disease to heart failure and its hypothetical change over time is examined by analyzing population trends in coronary disease, the data are difficult to reconcile with the aforementioned hypothesis of an increasing contribution of coronary disease to heart failure. Indeed, several groups reported on secular trends in the incidence of myocardial infarction indicating that, overall, the burden of incident hospitalized myocardial infarction, while displaced towards older age groups, is not increasing [107,108]. There is also evidence that the severity of myocardial infarction is decreasing [109] and that, consistent with these results, the incidence of heart failure after myocardial infarction is declining over time [110]. Taken collectively, these data are challenging to reconcile within the framework of the ongoing heart failure epidemic related to improved survival after myocardial infarction. While it is conceivable that more chronic forms of coronary disease could lead to heart failure without myocardial infarction, the role of chronic coronary disease in the genesis of heart failure is not defined. With regards to hypertension, conversely, unfavorable trends in awareness, treatment and control of hypertension have been documented [111,112]. Thus, coronary disease and hypertension trends in population studies both suggest that the attributable risk of hypertension for heart failure should remain high. To this end, Olmsted County data indicate that the risk of heart failure is greatest for coronary disease and diabetes, but that coronary disease and hypertension are responsible for the largest proportion of new heart failure cases in the population [113]. Over time, there was no evidence for a temporal change in the population attributable risk for heart failure of coronary disease, diabetes, and smoking. By contrast, the population attributable risk for hypertension increased from 15% (1979–1984) to 29% (1979–2002), and for obesity from 8% (1979–1984) to 17% (1997–2002) [113]. Indeed, the rising tide of diabetes mellitus [114] and obesity [115] raise the concern of an increasing role of these two entities in the genesis of heart failure. Notwithstanding uncertainties with regards to the exact cellular and molecular mechanisms by which obesity and diabetes impact both systolic and diastolic left ventricular function, there is mounting evidence for their causal link to heart failure independently of clinical coronary disease and hypertension [116–120]. Thus, the growing burden of diabetes and obesity in the population suggest that these two risk factors may be increasingly contributing to the heart failure epidemic.

## 7. Conclusion

Despite the staggering impact of heart failure as measured by Vital Statistics and administrative databases, validated longitudinal data on the incidence and outcome of heart failure remain sparse. The available data indicate that the incidence of heart failure is overall stable in the predominantly white populations in which it was studied.

Survival, which remains poor, is nevertheless improving over time. This results in an increase in the prevalence of heart failure and an increase in the number of individuals at risk for multiple hospitalizations. Future epidemiology research should investigate the heart failure epidemic in diverse populations and examine the burden and determinants of hospitalizations in heart failure.

## Figures and Tables

**Table 1.** Heart failure diagnostic criteria.

**Framingham** [3]	**Boston** [4]	**European Society of Cardiology** [60]	**Gothenburg Score** [5] **Item and method of assessment**	
**MAJOR CRITERIA** Paroxysmal nocturnal dyspnea or orthopnea Neck vein distension Rales Cardiomegaly Acute pulmonary edema S3 gallop Increased venous pressure ≥ 16cm water Circ.time ≥ 25 sec Hepatojugular reflux **MINOR CRITERIA** Ankle edema Night cough Dyspnea on exertion Hepatomegaly Pleural effusion Vital capacity decreased 1/3 from maximum Tachycardia rate of ≥ 120/min) **MAJOR OR MINOR CRITERION** Weight loss ≥ 4.5 kg in 5 days in response to treatment *HEART FAILURE present with 2 major or 1 major and 2 minor criteria*	**CATEGORY I: History** Rest dyspnea (4pts) Orthopnea (4pts) Paroxysmal nocturnal dyspnea (3 pts) Dyspnea on walking on level (2pts) Dyspnea on climbing (1pt) **CATEGORY II: Physical examination** Heart rate abnormality (1–2pts) Jugular venous pressure elevation (1–2 pts) Lung crackles (1–2pts) Wheezing (3 pts) Third heart sound (3 pts) **CATEGORY III: Chest radiography** Alveolar pulmonary edema (4 pts) Interstitial pulmonary edema (3 pts) Bilateral pleural effusions (3 pts) Cardiothoracic ratio ≥0.50 (3 pts) Upper-zone flow redistribution (2 pts) *Definite HEART FAILURE 8–12 pts, possible 5–7pts, unlikely 4 pts or less*	Symptoms of heart failure (at rest or during exercise) and Objective evidence of cardiac dysfunction (at rest) and Response to treatment directed towards heart failure (in cases where diagnosis is in doubt). *Criteria 1 and 2 should be fulfilled in all cases*	**CARDIAC SCORE**	
			History of heart disease (1–2pts)	Self-report
			Angina (1–2pts)	Self-report
			Edema (1pt)	Self-report
			Nocturnal Dyspnea (1pt)	Self-report
			Rales (1pt)	Physical exam
			Atrial fibrillation (1pt)	ECG
			**PULMONARY SCORE**	
			History of Chronic bronchitis/asthma (1–2pts)	Self-report
			Cough, phlegm, or wheezing (1pt)	Self-report
			Rhonchi (2pts)	Physical exam
			*Cardiac and pulmonary score are calculated and used to differentiate Cardiac form pulmonary dyspnea*	

**Table 2.** Cut-points used to define preserved ejection fraction in selected studies.

**Author, reference**	**Study**	**EF cut-off**
Yusuf, 2003 Lancet [121]	CHARM Preserved trial	40%
Lenzen, 2004 EHJ [122]	Euro HF survey	40%
Paulus 1998 EHJ [122]	EPICA Study	45%
Bhatia, 2006 NEJM [123]	EFFECT Study	>50 vs. <40
Zile, 2004 NEJM [28]	Multicenter study	>50%
Varadarajan, 2003 J Card Failure [124]	Single center hospital-based study	≥55%
Kitzman, J. AM. MED. ASSOC.2002 [25]	Cardiovascular Health Study	≥50%
Cortina, 2001 Am. J. Cardiol. [125]	Asturias	≥50%
Devereux 2000 Am. J. Cardiol. [97]	Strong Heart Study	>54%
Vasan 2000 Circulation [14]	Framingham Heart Study	>50%

**Table 3.** Selected studies reporting on the incidence and prevalence of heart failure (*denotes studies reporting on time trends).

**Author**	**Years**	**Incidence**	**Prevalence**	**Population source**	**Diagnostic criteria**
Gibson [126]	1962–64	---	1%	Rural US counties Whites All ages, not adjusted Survey of Physicians	No validation
Schocken [127]	1971–75	---	1-2%	NHANES I – Survey Ages 1–74 years, not adjusted	Self-report No validation
Senni [61]	1981, 1991	3/1000/yr	---	Olmsted County All ages, age adjusted Mostly whites	Framingham criteria
Ho [128]	1980’s	Women 1.4/1000/yr Men 2.3/1000/yr	0.8%	Framingham Heart Study Age adjusted	Framingham criteria
Croft* [62]	1986, 1993	White 24.6/1000/yr Black 26.1/1000/yr	---	Medicare enrollees First hospitalization Age adjusted	Discharge diagnoses No validation
Remes [129]	1986–88	Women 1.0/1000/yr Men 4.0/1000/yr	---	In and out patient national registries Age adjusted	Boston and Framingham criteria
Cowie [99]	1995–96	Women 1.2/1000/yr Men 1.4/1000/yr	---	Geographically defined in UK In- and out-patient All ages, not adjusted	European Society of Cardiology criteria
Gottdiener [101]	1990–96	Non black 19/1000/yr Black 19/1000/yr Women 15/1000/yr Men 26/1000/yr		Cardiovascular Health Study Age 65-100, age adjusted	Self-report Adjudication committee
Stewart* [63, 64]	1990–96	Women 1.3-1.9/1000/yr Men 1.27-2.2/1000/yr	---	Hospital discharge diagnoses All ages, not adjusted	No validation
Davies MK [130]	1995–99	---	2–3%	Random sample UK population Ages ≥ 45, not adjusted	European Society of Cardiology criteria
Nielsen OW [131]	1993–95	---	0.5–12%	General practice population Ages ≥ 50, not adjusted	Boston criteria
McCullough * [65]	1989–99	Women 3.7–4.2/1000/yr Men 4.0-3.7/1000/yr	Women 3.7–14.3/1000 Men 4.0–14.5/1000/	Henry Ford Health System 50% Whites Age adjusted	Framingham/NHANES in a sample
Levy* [68]	1950–99	∼5/1000/yr	---	Framingham Heart Study Age adjusted Mostly whites	Framingham criteria
Roger* [67]	1979–2000	∼3/1000/yr	---	Olmsted County Age adjusted Mostly whites	Framingham criteria
Barker [69]	1970–1974 ------------ 1990–1994	Women 8.6/1000/yr Men 11.7/1000/yr ------------------------- Women 11.8/1000/yr Men 12.7/1000/yr	---	Kaiser Permanente Age adjusted Mostly whites	Framingham criteria

**Table 4.** Selected studies on hospitalizations among patients with heart failure.

**Author**	**N**	**Year**	**Readmission**	**Readmission for heart failure**	**Data source**	**Definition criteria**	**Temporal trends**
McDermott [132]	612	1987–93		13% at 6 months	Hospital in Chicago	Dismissal diagnosis	No change
Krumholz [7, 133]	17,448	1991–94	44% at 6 months	---	Medicare files	DRG 127	---
Philbin EF [134]	2906	1995–97	43% at 6 months	---	Hospitals in New York	Admission and dismissal diagnoses	---
Cowie MR [135]	332	1996–97	59% at 19 months	---	Population-based in Scotland	European Society of Cardiology	---
Babayan [136]	493	1996–97	57% at 1 year	20% at 1 year	Johns Hopkins Hospital	DRG 127	---
Smith [137]	413	1996–98	46% at 6 months	19% at 6 months	New Haven hospital	Clinical	---
Baker [138]	22,203	1991–97	11% at 30 days	---	Medicare	ICD 9 codes	Increase over time
Lee WY [139]	1700	1999–2000	148 per 100 person-years	40 per 100 person-years	EPOCH Kaiser	Framingham	----
Lee DS [140]	77,421	1992–2002	---	27% at 1 year	Administrative database in Ontario	ICD 9 code 428	4 %/year decrease in 1 year
Badano [141]	179	1999–2000	48% at 6 months	---	Hospitals in Italy	Clinical	---
Rodriguez-Artalejo [142]	394	2000–2001	35% at 6 months	---	Hospitals in Spain	European Society of Cardiology	---
